# Natural light exposure and delirium in ICU: does the dark side cloud everything?

**DOI:** 10.1186/s13613-020-0643-3

**Published:** 2020-02-27

**Authors:** Romain Sonneville, Roland Smonig, Claire Dupuis, Lila Bouadma, Etienne de Montmollin, Jean-François Timsit

**Affiliations:** 1 Université de Paris, INSERM UMR1148, team 6, 75018 Paris, France; 2 Department of Intensive Care Medicine and Infectious Diseases, AP-HP, Bichat-Claude Bernard Hospital, 46 Rue Henri Huchard, 75018 Paris Cedex, France; 3 Medical Intensive Care Unit, Gabriel Montpied University Hospital, Clermont-Ferrand, France; 4 Université de Paris, INSERM UMR1137, team 5, 75018 Paris, France

## Letter to the editor

Dear Editor,

We read with interest the comments of Vahedian-Azimi and colleagues about our study [1]. In our study, antipsychotics (i.e., haloperidol) were given in 32/179 (18%) patients developing severe agitation during ICU stay. Unfortunately, doses of haloperidol were not part of study outcomes and were therefore not collected.

**Fig. 1:**
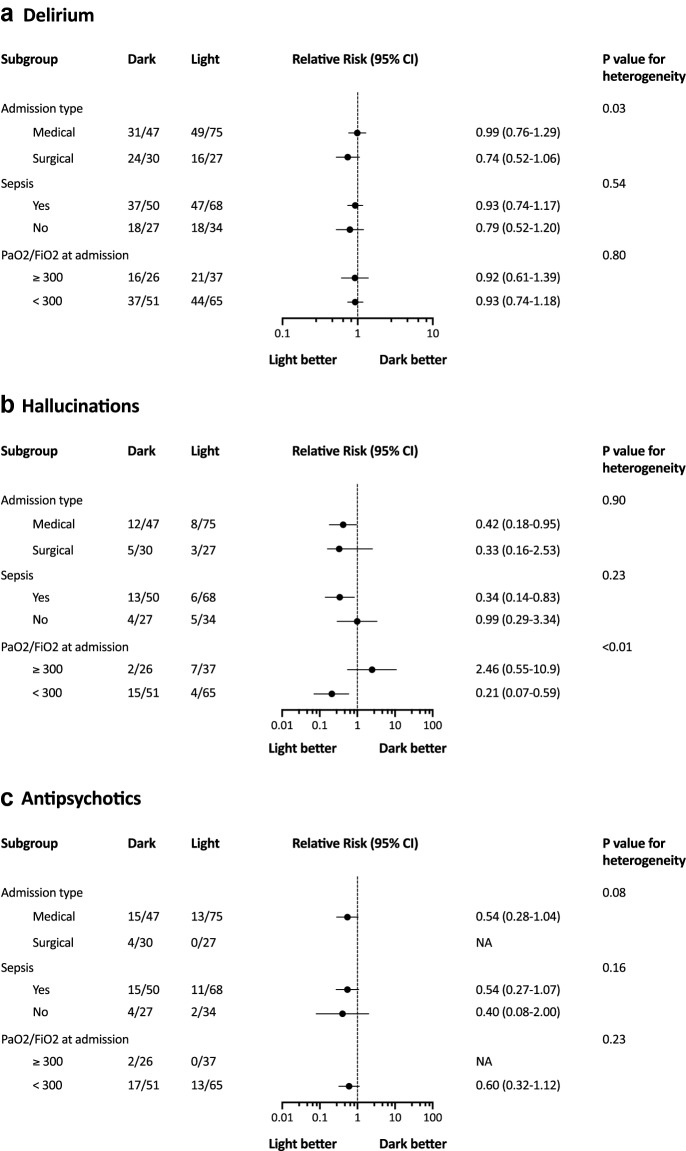
Effects of admission to a room with natural light exposure on delirium outcomes, post hoc subgroup exploratory analyses. **a** Delirium, measured on the intensive care delirium screening checklist (ICDSC); **b** hallucinations item of the ICDSC; **c** severe agitation episodes intervened with antipsychotics during ICU stay. LIGHT indicates admission to a room with natural light exposure via windows. DARK indicates admission to a room without natural light exposure. ICU intensive care unit

Based on a retrospective secondary analysis of a multicenter cohort of patients with acute respiratory distress syndrome, Vahedian-Azimi and colleagues report a higher delirium incidence in patients exposed only to artificial light during ICU stay, as compared to patients admitted to a room with windows, allowing exposure to natural light [2]. This association was observed both in crude analyses and after adjustment for common delirium risk factors. The authors accurately state that differences observed between their observations and others, including our study, may be explained by several methodological issues, likely contributing to outcome heterogeneity. Of note, reduced exposure to natural light may indeed be more detrimental in specific subgroups of patients, such as patients with ARDS or sepsis at admission, who often require a prolonged stay in the ICU. A previous study indeed suggested reduced mortality associated with natural light exposure via windows in medical patients with ICU stay greater than 72 h [3]. In our study, admission to a single room with potential exposure to natural light via windows (LIGHT group) was not associated with reduced delirium incidence, as compared to admission to a single room without windows (DARK group). However, natural light exposure was associated with a reduced risk of hallucinations and severe agitation episodes. A secondary exploratory analysis of our data in specific subgroups defined at ICU admission (i.e., medical admission, sepsis, and hypoxemia, defined by a PaO_2_/FiO_2_ ratio < 300) is presented in Fig. 1. In subgroup analyses, we found heterogeneity in the effect of LIGHT as compared with DARK on delirium between patients with different types of ICU admission. There was also heterogeneity in the effect of LIGHT as compared with DARK on hallucinations between patients with sepsis and patients without sepsis, and between patients with hypoxemia and patients without hypoxemia at ICU admission.

The effects of exposure to natural light via windows on delirium burden and outcomes may be clouded by several factors that should be systematically evaluated. These not only include patients’ characteristics and severity of illness at admission, but also environmental factors such as noise and interventions during ICU stay, including the use of sedative drugs and physical restraints. Moreover, illuminance in rooms may be dependent on daytime and seasons.

Further research evaluating the effect of natural light exposure in the ICU on outcomes should focus on delirium phenotypes and should also investigate other important patient-centered outcomes, such as anxiety/depression, functional recovery and ICU memories in survivors.

## Data Availability

The datasets used and/or analyzed during the current study are available from the corresponding author on reasonable request.
